# 1-Acetyl­oxymethyl-1,3,5,7-tetra­aza­adamantan-1-ium hexa­fluoro­phosphate

**DOI:** 10.1107/S1600536812017072

**Published:** 2012-04-21

**Authors:** Ming-Liang Liu

**Affiliations:** a Ordered Matter Science Research Center, Southeast University, Nanjing 211189, People’s Republic of China

## Abstract

In the crystal structure of the title salt, C_9_H_17_N_4_O_2_^+^·PF_6_^−^, the cations and anions are linked by weak C—H⋯F inter­actions while C—H⋯O inter­actions also occur between the cations.

## Related literature

The title compound was studied as part of a search for ferroelectric complexes. For background to ferroelectric complexes, see: Zhang *et al.* (2009 ▶, 2010 ▶); Ye *et al.* (2009 ▶). For a related structure, see: Reddy *et al.* (1994 ▶).
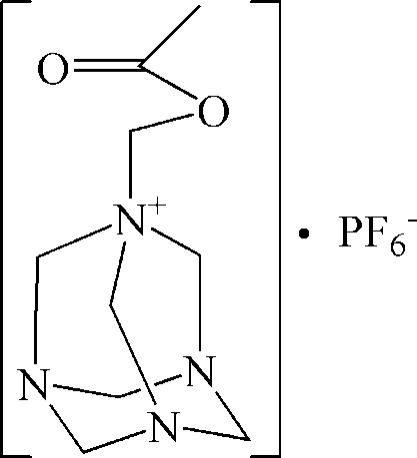


## Experimental

### 

#### Crystal data

C_9_H_17_N_4_O_2_^+^·PF_6_^−^
*M* *_r_* = 358.24
Monoclinic, 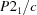

*a* = 8.2121 (16) Å
*b* = 15.697 (3) Å
*c* = 11.372 (2) Å
β = 90.26 (3)°
*V* = 1465.9 (5) Å^3^
*Z* = 4
Mo *K*α radiation
μ = 0.27 mm^−1^
*T* = 293 K
0.36 × 0.32 × 0.28 mm

#### Data collection

Rigaku Mercury2 diffractometer
Absorption correction: multi-scan (*CrystalClear*; Rigaku, 2005 ▶) *T*_min_ = 0.903, *T*_max_ = 0.921
14975 measured reflections
3358 independent reflections
2601 reflections with *I* > 2σ(*I*)
*R*_int_ = 0.035

#### Refinement

*R*[*F* ^2^ > 2σ(*F* ^2^)] = 0.093
*wR*(*F* ^2^) = 0.306
*S* = 1.06
3358 reflections
200 parameters
H-atom parameters constrained
Δρ_max_ = 1.00 e Å^−3^
Δρ_min_ = −0.66 e Å^−3^

### 

Data collection: *CrystalClear* (Rigaku, 2005 ▶); cell refinement: *CrystalClear*; data reduction: *CrystalClear*; program(s) used to solve structure: *SHELXTL* (Sheldrick, 2008 ▶); program(s) used to refine structure: *SHELXTL*; molecular graphics: *SHELXTL*; software used to prepare material for publication: *SHELXTL*.

## Supplementary Material

Crystal structure: contains datablock(s) I, global. DOI: 10.1107/S1600536812017072/xu5498sup1.cif

Structure factors: contains datablock(s) I. DOI: 10.1107/S1600536812017072/xu5498Isup2.hkl

Supplementary material file. DOI: 10.1107/S1600536812017072/xu5498Isup3.cml

Additional supplementary materials:  crystallographic information; 3D view; checkCIF report

## Figures and Tables

**Table 1:** Hydrogen-bond geometry (Å, °)

*D*—H⋯*A*	*D*—H	H⋯*A*	*D*⋯*A*	*D*—H⋯*A*
C1—H1*A*⋯F4^i^	0.97	2.42	3.370 (6)	167
C4—H4*B*⋯O2^ii^	0.97	2.59	3.441 (5)	147
C5—H5*A*⋯O2^ii^	0.97	2.47	3.350 (4)	151
C9—H9*C*⋯F4^i^	0.96	2.51	3.128 (8)	122

Symmetry codes: (i) 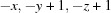; (ii) 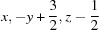.

**Fig. 1.**
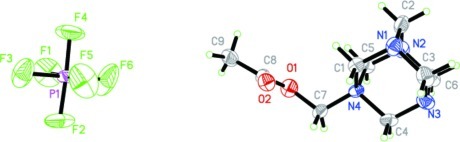
The molecular structure of the title compound, showing the atomic numbering scheme with 30% probability displacement ellipsoids.

## supplementary crystallographic information

### Comment

Recently much attention has been devoted to finding ferroelectric complexes. Ferroelectric materials that exhibit reversible electric polarization in response to an external electric field have found many applications such as nonvolatile memory storage, electronics and optics. The freezing of a certain functional group at low temperature forces significant orientational motions of the guest molecules and thus induces the formation of the ferroelectric phase (Zhang *et al.* 2009; Ye *et al.* 2009; Zhang *et al.* 2010). The title compound has been synthesized to investigate these properties.

There is a similar structure reported by Reddy *et al.* (1994).

The asymmetric unit of C_9_H_17_N_4_O_2_.PF_6_ consists of one 1-meyhyl acetate-1,3,5,7-tetra-aza-adamantan cation and one hexafluorophosphate anion linked by ionic bond (Fig 1). The hexafluorophosphate anion is a distorted octahedron. The P—F bonds are in the range 1.531 (4) to 1.559 (4) Å, the difference of the P—F bond distances are likely due to the different environment of F atoms. The bond angles around each phosphorus range from 84.3 (4)° to 179.1 (4)°. There is no classical hydrogen bond in the structure. The hexafluorophosphate anion is quite mobile, but examination of a difference map in the plane of the fluorine atoms does not show that fluorine atoms exist as three distinct atoms.

### Experimental

Hexamine, ammonium acetate and acetic anhydride were dissolved in water to give a solution refluxing at 373K, then hexafluorophosphoric acid was added to the above solution and filtered it. Single crystals suitable for X-ray structure analysis were obtained by the slow evaporation of the above solution after 10 days in air.

The dielectric constant of the compound as a function of temperature indicates that the permittivity is basically temperature-independent (*ε* = C/(T–T_0_)), suggesting that this compound is not ferroelectric or there may be no distinct phase transition occurring within the measured temperature range (below the melting point).

### Refinement

H atoms were placed in calculated positions with C—H = 0.96-0.97 Å, and refined in riding mode, U_iso_(H) = 1.2U_eq_(C) for methyl H atoms and 1.2U_eq_(C) for the others.

### Crystal data

**Table:**

C_9_H_17_N_4_O_2_^+^·PF_6_^−^	*F*(000) = 736
*M**_r_* = 358.24	*D*_x_ = 1.623 Mg m^−^^3^
Monoclinic, *P*2_1_/*c*	Mo *K*α radiation, λ = 0.71073 Å
Hall symbol: -P 2ybc	Cell parameters from 3026 reflections
*a* = 8.2121 (16) Å	θ = 3.4–26°
*b* = 15.697 (3) Å	µ = 0.27 mm^−^^1^
*c* = 11.372 (2) Å	*T* = 293 K
β = 90.26 (3)°	Block, colourless
*V* = 1465.9 (5) Å^3^	0.36 × 0.32 × 0.28 mm
*Z* = 4	

### Data collection

**Table:**

Rigaku Mercury2 diffractometer	3358 independent reflections
Radiation source: fine-focus sealed tube	2601 reflections with *I* > 2σ(*I*)
Graphite monochromator	*R*_int_ = 0.035
CCD_Profile_fitting scans	θ_max_ = 27.5°, θ_min_ = 3.2°
Absorption correction: multi-scan (*CrystalClear*; Rigaku, 2005)	*h* = −10→10
*T*_min_ = 0.903, *T*_max_ = 0.921	*k* = −20→20
14975 measured reflections	*l* = −14→14

### Refinement

**Table:**

Refinement on *F*^2^	Primary atom site location: structure-invariant direct methods
Least-squares matrix: full	Secondary atom site location: difference Fourier map
*R*[*F*^2^ > 2σ(*F*^2^)] = 0.093	Hydrogen site location: inferred from neighbouring sites
*wR*(*F*^2^) = 0.306	H-atom parameters constrained
*S* = 1.06	*w* = 1/[σ^2^(*F*_o_^2^) + (0.1929*P*)^2^ + 1.3689*P*] where *P* = (*F*_o_^2^ + 2*F*_c_^2^)/3
3358 reflections	(Δ/σ)_max_ = 0.002
200 parameters	Δρ_max_ = 1.00 e Å^−^^3^
0 restraints	Δρ_min_ = −0.66 e Å^−^^3^

### Special details

**Table:**

Geometry. All esds (except the esd in the dihedral angle between two l.s. planes) are estimated using the full covariance matrix. The cell esds are taken into account individually in the estimation of esds in distances, angles and torsion angles; correlations between esds in cell parameters are only used when they are defined by crystal symmetry. An approximate (isotropic) treatment of cell esds is used for estimating esds involving l.s. planes.
Refinement. Refinement of F^2^ against ALL reflections. The weighted R-factor wR and goodness of fit S are based on F^2^, conventional R-factors R are based on F, with F set to zero for negative F^2^. The threshold expression of F^2^ > 2sigma(F^2^) is used only for calculating R-factors(gt) etc. and is not relevant to the choice of reflections for refinement. R-factors based on F^2^ are statistically about twice as large as those based on F, and R- factors based on ALL data will be even larger.

### Fractional atomic coordinates and isotropic or equivalent isotropic displacement parameters (Å^2^)

**Table:**

	*x*	*y*	*z*	*U*_iso_*/*U*_eq_	
O2	0.3666 (3)	0.64498 (18)	0.9091 (2)	0.0560 (7)	
C9	0.1279 (6)	0.5718 (3)	0.8426 (5)	0.0723 (13)	
H9A	0.1479	0.5343	0.9078	0.108*	
H9B	0.1217	0.5392	0.7713	0.108*	
H9C	0.0269	0.6013	0.8544	0.108*	
C8	0.2634 (4)	0.6349 (2)	0.8339 (3)	0.0477 (8)	
C7	0.3995 (5)	0.7356 (3)	0.7032 (3)	0.0508 (9)	
H7A	0.4177	0.7324	0.6191	0.061*	
H7B	0.4964	0.7141	0.7422	0.061*	
O1	0.2653 (4)	0.6817 (2)	0.7323 (3)	0.0653 (8)	
P1	0.14024 (11)	0.21820 (7)	−0.00282 (9)	0.0520 (4)	
N4	0.3765 (3)	0.82704 (19)	0.7367 (2)	0.0405 (6)	
C5	0.2204 (4)	0.8657 (3)	0.6832 (3)	0.0504 (9)	
H5A	0.2214	0.8586	0.5985	0.060*	
H5B	0.1259	0.8361	0.7138	0.060*	
N1	0.3555 (5)	0.9303 (2)	0.8956 (3)	0.0600 (9)	
C1	0.3696 (4)	0.8413 (2)	0.8687 (3)	0.0454 (8)	
H1A	0.2770	0.8110	0.9009	0.054*	
H1B	0.4675	0.8187	0.9051	0.054*	
N2	0.2100 (4)	0.9539 (2)	0.7113 (4)	0.0623 (10)	
N3	0.5050 (4)	0.9668 (2)	0.7189 (4)	0.0631 (10)	
C4	0.5219 (4)	0.8786 (3)	0.6888 (4)	0.0536 (9)	
H4A	0.6225	0.8565	0.7218	0.064*	
H4B	0.5268	0.8726	0.6040	0.064*	
C3	0.4957 (6)	0.9756 (3)	0.8474 (4)	0.0657 (12)	
H3A	0.5947	0.9533	0.8826	0.079*	
H3B	0.4880	1.0355	0.8676	0.079*	
C2	0.2055 (6)	0.9639 (3)	0.8389 (5)	0.0673 (12)	
H2A	0.1117	0.9339	0.8697	0.081*	
H2B	0.1937	1.0238	0.8580	0.081*	
C6	0.3533 (6)	1.0000 (3)	0.6654 (5)	0.0736 (13)	
H6A	0.3429	1.0602	0.6829	0.088*	
H6B	0.3580	0.9935	0.5807	0.088*	
F2	0.2963 (5)	0.1801 (4)	−0.0600 (5)	0.1341 (17)	
F1	0.0194 (6)	0.1590 (4)	−0.0703 (4)	0.145 (2)	
F5	0.2628 (8)	0.2735 (3)	0.0688 (7)	0.165 (2)	
F3	0.1555 (5)	0.1442 (4)	0.0874 (5)	0.145 (2)	
F6	0.1267 (6)	0.2920 (5)	−0.0904 (7)	0.195 (3)	
F4	−0.0143 (6)	0.2520 (4)	0.0590 (7)	0.185 (3)	

### Atomic displacement parameters (Å^2^)

**Table:**

	*U*^11^	*U*^22^	*U*^33^	*U*^12^	*U*^13^	*U*^23^
O2	0.0519 (16)	0.0570 (16)	0.0589 (16)	−0.0045 (12)	−0.0119 (12)	−0.0019 (12)
C9	0.059 (3)	0.062 (3)	0.096 (4)	−0.017 (2)	−0.003 (2)	−0.010 (2)
C8	0.0404 (18)	0.0484 (19)	0.054 (2)	−0.0006 (14)	−0.0024 (15)	−0.0102 (15)
C7	0.049 (2)	0.056 (2)	0.0466 (19)	0.0045 (16)	0.0082 (15)	−0.0082 (16)
O1	0.0600 (18)	0.0689 (19)	0.0668 (19)	−0.0015 (15)	−0.0116 (14)	−0.0108 (15)
P1	0.0286 (5)	0.0715 (7)	0.0558 (6)	0.0050 (4)	0.0005 (4)	0.0015 (4)
N4	0.0283 (12)	0.0529 (16)	0.0401 (14)	0.0010 (11)	−0.0026 (10)	−0.0011 (12)
C5	0.0342 (16)	0.065 (2)	0.0520 (19)	−0.0025 (15)	−0.0137 (14)	0.0087 (16)
N1	0.058 (2)	0.0560 (19)	0.066 (2)	0.0003 (15)	−0.0060 (16)	−0.0143 (16)
C1	0.0434 (18)	0.055 (2)	0.0377 (16)	0.0010 (14)	−0.0031 (13)	−0.0049 (14)
N2	0.0427 (17)	0.057 (2)	0.088 (3)	0.0054 (14)	−0.0101 (16)	0.0096 (18)
N3	0.0454 (18)	0.062 (2)	0.082 (2)	−0.0096 (15)	−0.0068 (16)	0.0156 (18)
C4	0.0356 (17)	0.070 (2)	0.055 (2)	−0.0059 (16)	0.0024 (15)	0.0095 (18)
C3	0.062 (3)	0.057 (2)	0.079 (3)	−0.0122 (19)	−0.022 (2)	−0.005 (2)
C2	0.052 (2)	0.056 (2)	0.094 (3)	0.0108 (19)	0.003 (2)	−0.012 (2)
C6	0.059 (3)	0.066 (3)	0.097 (4)	−0.003 (2)	−0.014 (2)	0.028 (3)
F2	0.072 (2)	0.177 (4)	0.153 (4)	0.017 (3)	0.049 (2)	−0.030 (3)
F1	0.115 (3)	0.169 (5)	0.151 (4)	−0.014 (3)	−0.074 (3)	−0.038 (3)
F5	0.143 (5)	0.126 (4)	0.225 (6)	−0.028 (3)	−0.083 (4)	−0.046 (4)
F3	0.091 (3)	0.173 (5)	0.171 (4)	−0.015 (3)	−0.024 (3)	0.096 (4)
F6	0.100 (4)	0.235 (7)	0.250 (7)	−0.014 (4)	−0.024 (4)	0.177 (6)
F4	0.109 (4)	0.135 (4)	0.312 (8)	0.014 (3)	0.122 (5)	−0.044 (5)

### Geometric parameters (Å, º)

**Table:**

O2—C8	1.212 (5)	C5—H5A	0.9700
C9—C8	1.494 (5)	C5—H5B	0.9700
C9—H9A	0.9600	N1—C1	1.435 (5)
C9—H9B	0.9600	N1—C3	1.461 (6)
C9—H9C	0.9600	N1—C2	1.485 (6)
C8—O1	1.369 (5)	C1—H1A	0.9700
C7—O1	1.429 (5)	C1—H1B	0.9700
C7—N4	1.498 (5)	N2—C2	1.460 (6)
C7—H7A	0.9700	N2—C6	1.479 (6)
C7—H7B	0.9700	N3—C4	1.434 (6)
P1—F6	1.531 (4)	N3—C3	1.469 (6)
P1—F4	1.548 (4)	N3—C6	1.479 (6)
P1—F3	1.555 (4)	C4—H4A	0.9700
P1—F5	1.557 (4)	C4—H4B	0.9700
P1—F1	1.559 (4)	C3—H3A	0.9700
P1—F2	1.559 (3)	C3—H3B	0.9700
N4—C1	1.520 (4)	C2—H2A	0.9700
N4—C5	1.540 (4)	C2—H2B	0.9700
N4—C4	1.544 (4)	C6—H6A	0.9700
C5—N2	1.423 (6)	C6—H6B	0.9700
			
C8—C9—H9A	109.5	N4—C5—H5B	109.6
C8—C9—H9B	109.5	H5A—C5—H5B	108.1
H9A—C9—H9B	109.5	C1—N1—C3	109.2 (3)
C8—C9—H9C	109.5	C1—N1—C2	108.7 (3)
H9A—C9—H9C	109.5	C3—N1—C2	108.5 (4)
H9B—C9—H9C	109.5	N1—C1—N4	111.0 (3)
O2—C8—O1	121.0 (4)	N1—C1—H1A	109.4
O2—C8—C9	124.0 (4)	N4—C1—H1A	109.4
O1—C8—C9	115.1 (4)	N1—C1—H1B	109.4
O1—C7—N4	114.2 (3)	N4—C1—H1B	109.4
O1—C7—H7A	108.7	H1A—C1—H1B	108.0
N4—C7—H7A	108.7	C5—N2—C2	109.2 (3)
O1—C7—H7B	108.7	C5—N2—C6	110.4 (4)
N4—C7—H7B	108.7	C2—N2—C6	108.7 (4)
H7A—C7—H7B	107.6	C4—N3—C3	109.5 (3)
C8—O1—C7	121.7 (3)	C4—N3—C6	108.9 (4)
F6—P1—F4	88.8 (4)	C3—N3—C6	109.2 (4)
F6—P1—F3	179.1 (4)	N3—C4—N4	110.2 (3)
F4—P1—F3	91.2 (4)	N3—C4—H4A	109.6
F6—P1—F5	87.9 (4)	N4—C4—H4A	109.6
F4—P1—F5	95.8 (4)	N3—C4—H4B	109.6
F3—P1—F5	91.3 (4)	N4—C4—H4B	109.6
F6—P1—F1	95.0 (4)	H4A—C4—H4B	108.1
F4—P1—F1	84.6 (3)	N1—C3—N3	111.9 (3)
F3—P1—F1	85.9 (3)	N1—C3—H3A	109.2
F5—P1—F1	177.1 (3)	N3—C3—H3A	109.2
F6—P1—F2	94.4 (4)	N1—C3—H3B	109.2
F4—P1—F2	176.9 (4)	N3—C3—H3B	109.2
F3—P1—F2	85.7 (3)	H3A—C3—H3B	107.9
F5—P1—F2	84.3 (4)	N2—C2—N1	111.7 (3)
F1—P1—F2	95.1 (3)	N2—C2—H2A	109.3
C7—N4—C1	113.5 (3)	N1—C2—H2A	109.3
C7—N4—C5	112.5 (3)	N2—C2—H2B	109.3
C1—N4—C5	107.3 (3)	N1—C2—H2B	109.3
C7—N4—C4	108.3 (3)	H2A—C2—H2B	107.9
C1—N4—C4	107.7 (3)	N2—C6—N3	110.6 (4)
C5—N4—C4	107.4 (3)	N2—C6—H6A	109.5
N2—C5—N4	110.2 (3)	N3—C6—H6A	109.5
N2—C5—H5A	109.6	N2—C6—H6B	109.5
N4—C5—H5A	109.6	N3—C6—H6B	109.5
N2—C5—H5B	109.6	H6A—C6—H6B	108.1

### Hydrogen-bond geometry (Å, º)

**Table:**

*D*—H···*A*	*D*—H	H···*A*	*D*···*A*	*D*—H···*A*
C1—H1*A*···F4^i^	0.97	2.42	3.370 (6)	167
C4—H4*B*···O2^ii^	0.97	2.59	3.441 (5)	147
C5—H5*A*···O2^ii^	0.97	2.47	3.350 (4)	151
C9—H9*C*···F4^i^	0.96	2.51	3.128 (8)	122

Symmetry codes: (i) −*x*, *y*+3/2, −*z*+3/2; (ii) −*x*, −*y*+2, −*z*.

## References

[bb1] Reddy, D. S., Panneerselvlvam, K., Shimoni, L., Carrell, H. L. & Desiraju, G. R. (1994). *J. Mol. Struct.* **327**, 113–120.

[bb2] Rigaku (2005). *CrystalClear* Rigaku Corporation, Tokyo, Japan.

[bb3] Sheldrick, G. M. (2008). *Acta Cryst.* A**64**, 112–122. 10.1107/S0108767307043930 18156677

[bb4] Ye, H.-Y., Fu, D.-W., Zhang, Y., Zhang, W., Xiong, R.-G. & Huang, S.-P. (2009). *J. Am. Chem. Soc.* **131**, 42–43. 10.1021/ja808331g 19128170

[bb5] Zhang, W., Chen, L.-Z., Xiong, R.-G., Nakamura, T. & Huang, S.-P. (2009). *J. Am. Chem. Soc.* **131**, 12544–12545. 10.1021/ja905399x 19685869

[bb6] Zhang, W., Ye, H.-Y., Cai, H.-L., Ge, J.-Z., Xiong, R.-G. & Huang, S.-P. (2010). *J. Am. Chem. Soc.* **132**, 7300–7302. 10.1021/ja102573h 20459097

